# Dendritic Cells Generated From *Mops condylurus*, a Likely Filovirus Reservoir Host, Are Susceptible to and Activated by Zaire Ebolavirus Infection

**DOI:** 10.3389/fimmu.2019.02414

**Published:** 2019-10-11

**Authors:** Kathryn M. Edenborough, Marcel Bokelmann, Angelika Lander, Emmanuel Couacy-Hymann, Johanna Lechner, Oliver Drechsel, Bernhard Y. Renard, Aleksandar Radonić, Heinz Feldmann, Andreas Kurth, Joseph Prescott

**Affiliations:** ^1^Centre for Biological Threats and Special Pathogens, Robert Koch Institute, Berlin, Germany; ^2^LANADA, Laboratoire National d'Appui au Développement Agricole, Bingerville, Côte d'Ivoire; ^3^Methodology and Research Infrastructure, Robert Koch Institute, Berlin, Germany; ^4^Laboratory of Virology, Division of Intramural Research, National Institute of Allergy and Infectious Diseases, National Institutes of Health, Hamilton, ON, United States

**Keywords:** Ebola virus (EBOV), reservoir hosts, dendritic cells, transcriptome, *Mops condylurus*, filovirus

## Abstract

Ebola virus infection of human dendritic cells (DCs) induces atypical adaptive immune responses and thereby exacerbates Ebola virus disease (EVD). Human DCs, infected with Ebola virus aberrantly express low levels of the DC activation markers CD80, CD86, and MHC class II. The T cell responses ensuing are commonly anergic rather than protective against EVD. We hypothesize that DCs derived from potential reservoir hosts such as bats, which do not develop disease signs in response to Ebola virus infection, would exhibit features associated with activation. In this study, we have examined *Zaire ebolavirus* (EBOV) infection of DCs derived from the Angolan free-tailed bat species, *Mops condylurus*. This species was previously identified as permissive to EBOV infection *in vivo*, in the absence of disease signs. *M. condylurus* has also been recently implicated as the reservoir host for *Bombali ebolavirus*, a virus species that is closely related to EBOV. Due to the absence of pre-existing *M. condylurus* species-specific reagents, we characterized its *de novo* assembled transcriptome and defined its phylogenetic similarity to other mammals, which enabled the identification of cross-reactive reagents for *M. condylurus* bone marrow-derived DC (bat-BMDC) differentiation and immune cell phenotyping. Our results reveal that bat-BMDCs are susceptible to EBOV infection as determined by detection of EBOV specific viral RNA (vRNA). vRNA increased significantly 72 h after EBOV-infection and was detected in both cells and in culture supernatants. Bat-BMDC infection was further confirmed by the observation of GFP expression in DC cultures infected with a recombinant GFP-EBOV. Bat-BMDCs upregulated CD80 and chemokine ligand 3 (CCL3) transcripts in response to EBOV infection, which positively correlated with the expression levels of EBOV vRNA. In contrast to the aberrant responses to EBOV infection that are typical for human-DC, our findings from bat-BMDCs provide evidence for an immunological basis of asymptomatic EBOV infection outcomes.

## Introduction

Ebola virus [(EBOV) species *Zaire ebolavirus*, Filoviridae], has jumped the species barrier resulting in more than 15 separate human outbreaks since its discovery in the Democratic Republic of Congo (DRC), formerly Zaire, in 1976 (1). Ebola virus disease (EVD) in humans is characterized by a sudden onset of fever, malaise, chills, headache, myalgia, which can progress to severe gastrointestinal, respiratory, and neurological symptoms, haemorrhagic fever, meningoencephalitis, pancreatitis, hepatic and renal injury [reviewed in (2, 3)]. Fatality rates are strain-dependent and range between 40 and 90% with many patients succumbing to internal hemorrhaging or multiple organ failure (1) partially as a result of aberrant cytokine and chemokine production (4) and impaired adaptive immune responses (5).

Significant inroads into controlling EBOV outbreaks have been made, including the development and implementation of ring vaccination methodologies [reviewed in (6)]. Despite this, in areas where public health responses are hindered such as the DRC 2018-9 Kivu outbreak, identification of reservoir hosts may assist in preventing spillover events. Our understanding of pathogenic filovirus reservoirs is founded on Marburg virus (MARV), a related filovirus, and its reservoir host the Egyptian rousette bat, *Rousettus aegyptiacus* (7). MARV was directly isolated from cave-dwelling *R. aegyptiacus*, which were encountered by humans who later developed MARV haemorrhagic fever (8). MARV isolates from this colony successfully reinfect bats upon experimental infection (7).

Unlike MARV, infectious EBOV has never been isolated from bats, however numerous human index cases are reported to have contacted bats prior to developing EVD (9, 10). In the field, EBOV-specific antibodies and RNA have been detected in serum and tissue samples from healthy bats, with a predominant sampling of fruit bats (11, 12). A pioneering study has shown that not only fruit bats but also the insectivorous bat, *M. condylurus* can be experimentally infected with EBOV (13). Not only have EBOV-specific antibodies been detected in wild populations of this species but it is also considered as the source of the 2014 outbreak in West Africa as a result of suspected exposure of an index case to a colony (9). In addition, viral genomic sequence of Bombali virus, a newly discovered ebolavirus species, has been detected in swab (14) and tissue samples at high vRNA levels from wild *M. condylurus* (15). This collective information provides conclusive evidence that *M. condylurus* plays a considerable role in EBOV ecology.

Studies examining the EBOV infection potential in bats have focussed on the susceptibility of bat derived fibroblast or epithelial cell cultures to infection *in vitro* (16, 17). However, it is also necessary to study cell types that are key to disease exacerbation in humans, such as DCs and macrophages as their aberrant responses to EBOV infection have been implicated in contributing to EVD (18, 19). Macrophages support EBOV replication and are thought to contribute to inflammation and haemorrhagic fever syndrome via excessive cytokine release and production of reactive oxidative species (20–24). While DCs also support EBOV replication, they remain in a state of paralysis depicted by *in vitro* studies where suppression of surface expressed maturation markers such as CD80, CD86, and MHC class II molecules post-infection have been observed paralleled with the upregulation of T cell inhibitory molecules such as B7-H1 resulting in PD1 mediated T cell apoptosis (25–27).

In this study, we generated and interrogated the *de novo* assembled transcriptome for *M. condylurus* and identified immunological reagents to study the susceptibility and immune response of their BMDCs to EBOV infection. We demonstrated that bat-BMDCs are susceptible to EBOV infection, which is akin to findings of past studies that also outline the permissiveness of human and non-human primate (NHP) monocyte derived DC to infection. Unlike the antiviral responses of human and NHP DC to EBOV infection, which are marked by functional impairment and suppression, we found a feature of the bat-BMDC response to EBOV was upregulation of the activation-marker CD80 and chemokine CCL3 transcripts, which both correlated with vRNA amplification. The susceptibility and antiviral responses of *M. condylurus* DCs to EBOV infection further support its status as a reservoir host for Ebolavirus and provide insight into immunological features of Ebola virus infection in a reservoir host species.

## Results

### Assembly and Analysis of *M. condylurus* Transcriptome

To identify reagents that could be used to characterize microbat immune responses to EBOV infection, RNA from *M. condylurus* was sequenced to compile a *de novo* assembled transcriptome that contained 547,036 contiguous sequences (contigs) (Supplementary Table 1). After filtering with Transrate, the Uniprot database annotated 80,761 contigs confined to 50,691 genes that had various isoforms associated with each annotation. The median contig length was 888 nucleotides and the assembly N50 length was 2,345 nucleotides with a GC content of 46%. Using the Benchmarking Universal Single-Copy Ortholog (BUSCO) analysis tool we determined 91.1% complete benchmarking orthologs were detected in the *M. condylurus* assembly while 7.2% were fragmented and 1.3% were missing.

**Figure 1:**
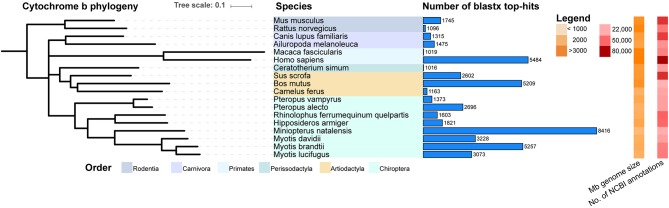
*Mops condylurus* transcriptome top-hits to *Mammalia* in NCBI database. MrBayes phylogenetic tree for the cytochrome B gene was built with Geneious (v10.0.5) on ClustalW aligned genes. Contigs generated via *de novo* assembly of *M. condylurus* sequences were subjected to blast2go analysis against the GenBank Mammalia database. For each contig a top-hit (blastx match with the lowest e-value) was determined and the number of contigs, for species with more than 1000 top-hits, is represented with horizontal light-blue bars. The order classification for each species is indicated by colored panels. The genome size in megabases and number of annotated mRNAs present in GenBank, at the time of analysis, are displayed as orange and red heat maps, respectively. This figure was generated with interactive tree of life (iTOLv1).

In order to compare the sequence similarity of these identified contigs to sequences within Mammalia in GenBank, the 80,761 contigs were subjected to a blast2go analysis. Only BLAST hits with the lowest e-value (top-hits) were considered in the species analysis to prevent one contig matching to multiple species. By implementing the query coverage statistics (qcovs) we calculated 9,316 contigs had more than 80% of their length aligned to top-hit subject sequences (Supplementary Table 1). The top-hit counts from this analysis are shown for species present within the Mammalia database having more than 1,000 hits detected (Figure 1). To illustrate relatedness between the species, a phylogenetic tree for the cytochrome b gene is also depicted vertically in an iTOL tree diagram. A high number of top-hits matched to species within the Chiroptera order, including both macrobats and microbats, albeit contigs blasted more often to the latter, including *Myotis davidii, Myotis brandtii, Myotis lucifugus*, and with the highest number of related hits to *Miniopterus natalensis*. Interestingly, *M. condylurus* contigs matched to a high number of human, *Sus scrofa* (pig) and *Bos mutus* (yak) sequences, despite being phylogenetically distant to these mammals.

### The Identification and Validation of Cross-Reactive Antibodies to Phenotype *M. condylurus* Immune Cells

**Figure 2:**
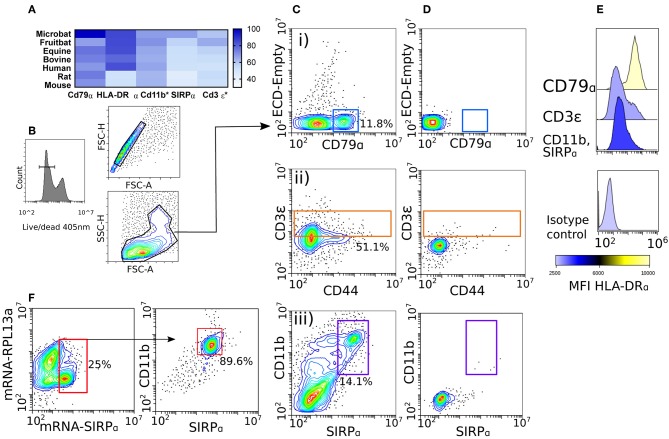
Phenotyping splenocytes with cross-reactive antibody panels. Protein homology assessment was performed by ClustalW alignment of *M. condylurus* and Uniprot sequences belonging to the following species; *Myotis brandtii* (Microbat), *Pteropus alecto* (Fruitbat), *Equus caballus* (Equine), *Bos taurus* (Bovine), *Homo sapiens* (Human), *Rattus norvegicus* (Rat), and *Mus musculus* (Mouse). In **(A)** the alignment is depicted as a heat map for immunological markers that are conserved across species and used in immune cell phenotyping for this study. *Indicates proteins where only a partial open reading frame was available from the transcriptome for alignment. Splenocytes were collected from *M. condylurus* and stained with viable cell dyes **(B)**, cross-reactive antibodies targeting intracellular markers (Cd79α and Cd3ε) or surface markers (CD44, Cd11b, and SIRPα) **(C)** and isotype controls **(D)**. An antibody targeting HLA-DRα was used to measure MHC class II expression on various gated populations **(E)**. PrimeFlow™ probes **(F)** were designed for less-conserved molecules to correlate antibody binding and mRNA expression.

We sought to identify conserved cell markers expressed by immune cells by comparing amino acid percent identities between diverse mammals and *M. condylurus*, presented as a heatmap in Figure 2A. Our analyses revealed CD79α, HLA-DRα, and CD11b of *M. condylurus* shared >75% identity with amino acid sequences of other species for which antibodies are already commercially available (i.e., human, mouse and rat). The high degree of homology observed was used to select antibody clones that allow us to distinguish T cells and B cells based on CD3ε^+^ and CD79α^+^ expression, respectively, in concert with their reciprocal expression of MHC Class II molecules (HLA-DRα).

Antibody validation was performed using *M. condylurus* splenocytes as this lymphoid organ has high lymphocyte and antigen presenting cell (APC) numbers. Our gating strategy (Figure 2B) identified a population of cells expressing CD79α (Figure 2Ci). Expression levels of antigens detected using these antibodies were well above that when an isotype control was used (Figure 2D). These cells were also found to express high levels of MHC Class II (Figure 2E) and are in line with expected levels of CD79α^+^MHC Class II^hi^ B cells in other species (28). To distinguish CD3ε^+^ T cells, we also included an antibody for the activation marker CD44 (Figure 2Cii). Although no CD44 contig was detected in the *M. condylurus* transcriptome, we were able to identify a notable population of CD3ε^+^ cells with varying levels of CD44 which may reflect different activation states (29). These cells were found to express lower levels of MHC Class II than the CD79α^+^ population befitting of CD3ε^+^MHC Class II^lo−int^ expression levels, which can be very diversely regulated on T cells in other species (Figure 2E) (30).

We verified the use of known DC-associated markers, SIRPα, CD11b, and MHC Class II (31). For SIRPα, which is less conserved between *M. condylurus* and other species examined (Figure 2A), labeled mRNA probes were designed and utilized in Prime Flow assays to correlate specific RNA expression with the binding of a candidate anti-SIRPα antibody. At the same time a probe designed to detect the housekeeping gene RPL13 was used to ensure probe hybridization was successful. Our results show that ~25% of the total splenocyte population expressed SIRPα mRNA (Figure 2F). Ninety percent of these SIRPα mRNA expressing cells were also found to co-express surface SIRPα as well as CD11b using specific antibodies for these markers. The high degree of correlation exhibited by the SIRPα mRNA probe and antibody binding therefore indicates that bona fide SIRPα is expressed in concert with CD11b^+^ and represents a viable APC phenotype in *M. condylurus*. To examine the frequency of SIRPα^+^CD11b^+^ APCs within a *M. condylurus* splenocyte population, we gated out T and B cells, revealing a population of CD79α^−^CD3ε^−^SIRPα^+^CD11b^+^ cells (Figure 2Ciii) that exhibited intermediate expression levels of MHC Class II (Figure 2E).

**Figure 3:**
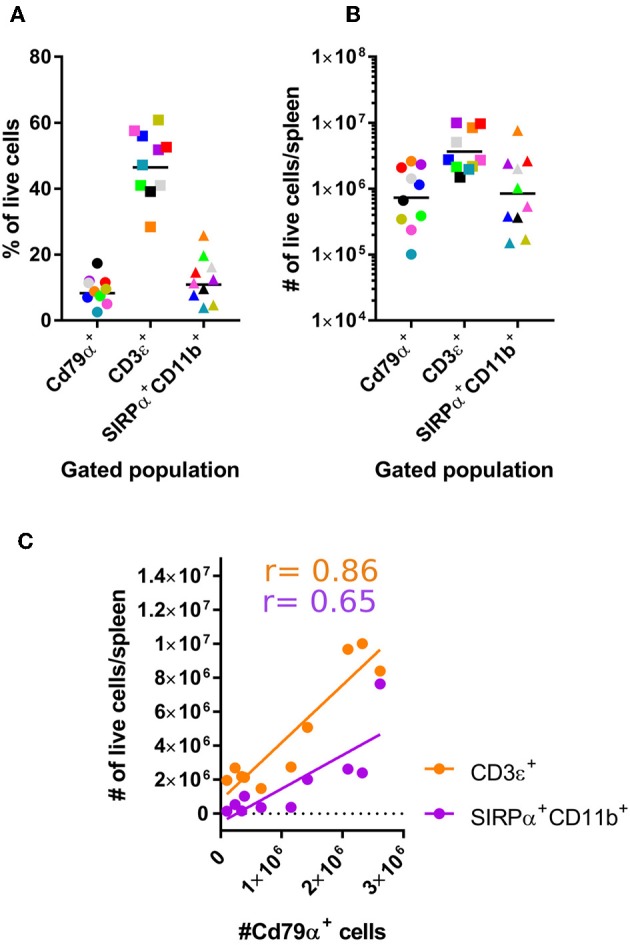
Enumerating lymphocyte and DC populations in *M. condylurus* spleen. Single cell suspensions were prepared from spleen and stained with the antibody panels indicated on the x-axis in **(A)** and the percentage of Cd79α^+^, CD3ε^+^, and SIRPα^+^CD11b^+^ cells from 10 individual *M. condylurus* bats are shown. Cell number was calculated according to (% of marker positive population × [% viable cells x (total cell count)]-number of marker positive cells identified in isotype control stained samples) × total volume spleen **(B)**. An xy plot correlating CD79α^+^ cell number with CD3ε^+^ and SIRPα^+^CD11b^+^ cell numbers is presented **(C)** with Pearson correlation *r* values.

Using these established antibody panels, we examined the frequencies of splenic SIRPα^+^CD11b^+^ APCs, CD3ε^+^ T, and CD79α^+^ B cells harvested from a larger sample size of bats. Our results showed T cell frequencies to be greater than APCs and B cells (Figure 3A). A similar trend was also evident when cells of each population are expressed as viable cell number/spleen (Figure 3B). Of note, we found that percentages of APCs, B cells, T cells to be relatively proportional to each other within each animal (Figure 3C). For example, animals with lower percentages of CD79α^+^ B cells in spleen also appeared to have lower percentages of CD3ε^+^ T cells and SIRPα^+^CD11b^+^ APCs.

### *M. condylurus* Phylogeny of GM-CSF and IL-4

A phylogenetic analysis of IL-4 and GM-CSF sequences from *M. condylurus* and mammals for which a number of immunological reagents are available were undertaken to determine homologous proteins that could promote DC differentiation in *M. condylurus*. ClustalW alignment of these sequences from *M. condylurus* with numerous mammals suggests that murine or human GM-CSF or IL-4 would not be suitable for DC differentiation due to the large phylogenetic distances, however, proteins with greater homology including those from equine or bovine species would be ideal for propagating bat-BMDC cultures (Supplementary Figures 2A,B).

### Morphology of *M. condylurus* BMDCs Generated *in vitro* in the Presence of Equine GM-CSF and IL-4

**Figure 4:**
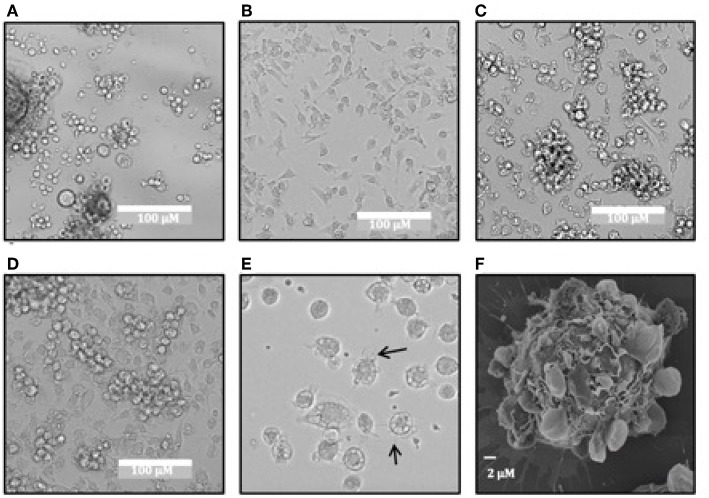
Morphology of *M. condylurus* BMDCs. Dendritic cell morphology of bat bone-marrow cultures is shown on days 5 for cultures supplemented with mouse GM-CSF/IL4 **(A)** or equine GM-CSF/IL4 **(B)** and on days 7 **(C)** and 9 **(D)** and 10 **(E)** for cultures supplemented with equine GM-CSF/IL4. SEM images of DC cultures were taken on day 10 of culture at 5000x **(F)** magnification.

While evaluating the use of recombinant equine IL-4 and GM-CSF in *M. condylurus* for bone marrow-derived cultures, we observed cells with morphological characteristics consistent with DCs including formation of a stromal cell monolayer (Figure 4B) and semi-adherent clusters over the course of 6–9 days (Figures 4C,D). This cell differentiation was not observed in cultures supplemented with murine GM-CSF and IL-4 (Figure 4A). After 6–10 days punctate veiled spherical cells possessing motile cytoplasmic projections were also observed (Figure 4E). While the cultures were heterogenous, potentially containing macrophages, the predominant cell type identified displayed distinct motile cytoplasmic projections (Supplementary Video 1), which is a feature consistent with DC types rather than macrophages (32). DC-like cells from the spleen could also be propagated using this same method but was found to yield lower morphologically-similar cell numbers (data not shown). Examination of bat-BMDC cultures on day 10 with scanning electron microscopy revealed predominant round cells with lamellar and filamentous surface structures (Figure 4F).

### Phenotype and Maturation of *in vitro* Cultured *M. condylurus* BMDCs

**Figure 5:**
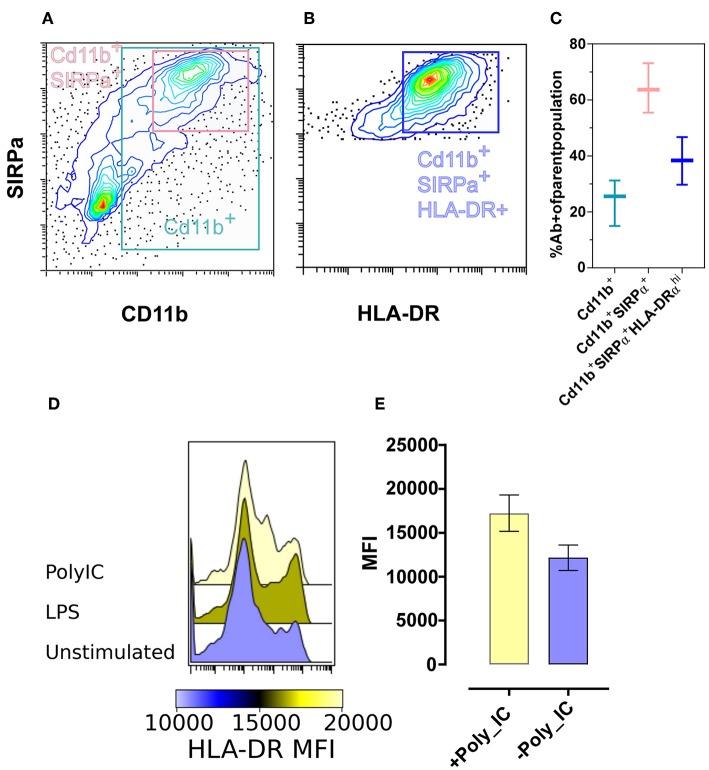
Phenotype and reactivity of *M. condylurus* BMDCs. BMDC phenotype CD3ε-, CD79α-, Cd11b^+^ was measured with flow cytometry. BMDC cultures stained with the markers Cd11b^+^, SIRPα^+^, and HLA-DRα^+^ are portrayed in contour plots **(A,B)** and the percent of each cell type from cultures of three different wild caught *M. condylurus* are shown **(C)**. BMDC cultures at day 6 were treated with or without Poly IC and LPS and the median fluorescence intensity for HLA expression of SIRPα^+^ gated cells are shown in **(E)** for three separate cultures with standard error and representative histograms **(D)**.

We next applied the use of the antibody panels to profile bat-BMDC cultures. By first gating out CD3ε^+^ T and CD79α^+^ B cells, we found that around 30% of remaining cells were CD11b^+^ and within this population, ~70% were SIRPα^+^ (Figure 5A). Further analysis of these CD3ε^−^CD79α^−^ SIRPα^+^CD11b^+^ cells revealed ~40% expressed detectable surface levels of MHC class II (Figure 5B). These percentages were largely consistent across cultures derived from different individual bats with the coefficient of variation observed in each cell population falling between 13 and 34% (Figure 5C).

In evaluating the functional capacity of these cells to respond to a TLR agonist, we measured the regulation of surface expression of MHC Class II following incubation with LPS and poly IC. Compared to unstimulated cells, of which SIRPα+ live gated population had a median fluorescence intensity (MFI) of 12,750 for MHC class II expression, cells treated with poly IC led to increase in levels of MHC class II (Figures 5D,E). Thus, not only do these propagated cells exhibit a similar APC-like phenotype as that shown for spleen based on their expression of SIRPα and CD11b but have an intermediate-high MHC class II expression level consistent with defined DC phenotypes for other species and are also activated by typical DC agonists.

### EBOV Infection of *M. condylurus* BMDCs and Their Chemokine and Cytokine Responses

**Figure 6:**
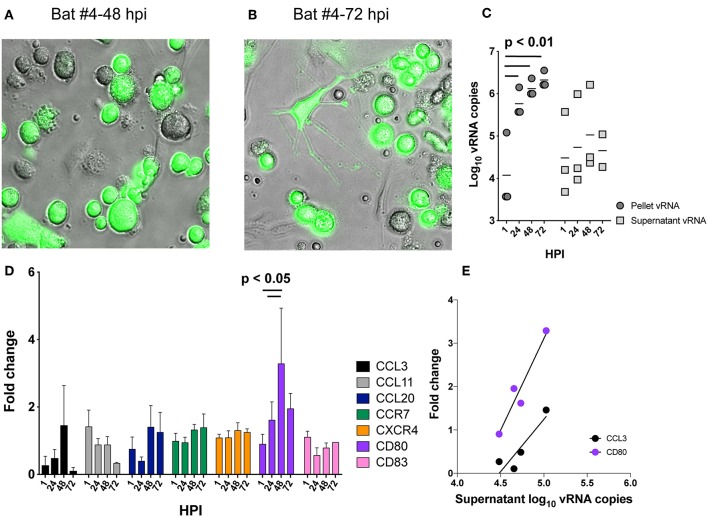
Response of bat-BMDC to EBOV infection. BMDC cultures from bat#4 were infected with GFP-tagged EBOV and cultures were visualized with an EVOS FL Imaging System at 48 **(A)** and 72 h post infection (HPI) **(B)**. Day 6–10 BMDC cultures from three separate bats (#3, #4, and #43) were inoculated with EBOV at an MOI of 3 for 1 h and supernatant and cell pellets were collected at 1, 24, 48, and 72 hpi and subjected to RNA extraction to quantify viral copies. The log_10_ vRNA copies/100 μl of supernatant (dark-gray circles) or cell pellet (light-gray squares) are shown for each bat-BMDC culture individually **(C)**. RNA isolated from cell pellet was further subjected to qPCR assays measuring host transcripts and the relative fold changes in gene expression are shown for CCL3, CCL11, CCL20, CCR7, CXCR4, CD80, and CD83 in **(D)**, a bar graph showing mean values and standard deviation for the three bat-BMDC cultures tested. Relative fold change was calculated by (gene absolute copy number in infected cultures/gene absolute copy number in matched and uninfected BMDC cultures) where all values were normalized to the housekeeping gene RPL13. Significant correlations between viral RNA copies and fold change in host gene expression was determined by Pearsons test and the linear regression defining the correlations are shown in an xy plot **(E)**. Mixed-effects model with Tukey multiple comparisons posttest determined significance in **(C,D)**.

To ascertain whether bat-BMDCs are permissive to EBOV infection *in vitro*, five separate cultures derived from bat #3, #4, #42, #43, and #44 were differentiated with IL-4 and GM-CSF for 6–10 days and then inoculated with EBOV-Zaire to monitor the development of infection. One culture was inoculated with GFP-tagged EBOV to visualize the cell types susceptible to infection, which demonstrated numerous infected cells display a DC-like morphology (Figures 6A,B), suggesting viral genome amplification and host responses result from DCs, rather than from stromal cell infection.

All of the five cultures were inoculated with EBOV for 1 h and RNA was extracted from culture supernatants and cell pellets after 1, 24, 48, and 72 h and EBOV genome copies quantitated by one-step reverse transcription-qPCR (RT-qPCR). EBOV genome amplification was detected in culture supernatants and cell pellets from three bats (#3, #4, and #43) of the five inoculated. In the cultures from the other two bats (#42 and #44) no increase in genome amplification over 72 h post infection (HPI) was detected (data not shown). These cultures did not expand in the presence of GM-CSF and IL4 and were likely non-viable, hence these samples were excluded from further analyses.

For the three EBOV-susceptible bat-BMDC cultures, the vRNA copies detected in supernatant and cell pellets increased over time as significantly more vRNA copies were detected at the 24, 48, and 72 h timepoints than 1 HPI (*p* < 0.01, Tukey's multiple comparison test) (Figure 6C). Compared to the number of vRNA copies detected from cell pellets, more variation was apparent in supernatant vRNA levels between BMDC cultures derived from different bats (Figure 6C). Despite this, the levels of vRNA increased in supernatants within each bat-BMDC culture. The average fold increase of vRNA detected in supernatants was 1.35 log_10_ ± 0.298 SD between 1 and 72 HPI.

To determine if enhanced transcription of antiviral-associated genes in bat-BMDC cultures was induced by EBOV infection, multiplex one-step RT-qPCR assays were designed to detect genes previously shown to be upregulated in human DCs and macrophages during infection with EBOV virus like particles (VLPs) or during Reston EBOV infection (33). The genes encoded for chemokines: CCL20 (MIP3α), CCL11 (eotaxin1), CCL3 (MIP1α) and activation markers: CCR7, CD80, CD83 and as an extraction control a housekeeping gene was assessed: RPL13. The efficiency curves and primer sequences for each target are presented in Supplementary Table 2.

qPCR assays using these primers were performed on RNA extracted from cell pellets following infection to detect host specific differences in gene expression in bat-BMDCs. The expression levels for CCL3, CCL11, CCL20, CCR7, CXCR4, and CD83 transcripts did not increase significantly in response to EBOV infection (Figure 6D). CD80 expression was significantly upregulated at 24 and 48 HPI in comparison to baseline expression levels at 1 HPI.

The expression levels varied greatly for some transcripts between individual bats. To develop a more detailed understanding of transcript expression over time for each target we correlated fold change with vRNA copies expressed over the 72 h of infection (Figure 6E). Increase in vRNA copies for BMDC cultures derived from bats #3, #4, and #43 positively correlate with increase in expression of two host transcripts; CD80 and CCL3 (Pearson's *r* = 0.020 and 0.049, respectively). As matched, uninfected BMDC cultures from each individual bat are used to calculate fold change in gene expression for each timepoint, the increase in CD80 and CCL3 transcription coincided only with viral infection.

## Discussion

*Mops condylurus* has been postulated as an Ebola virus reservoir host due to; its susceptibility to experimental EBOV infection (13), positive detection of EBOV specific IgG in serum from wild *M. condylurus* (34), association with the 2014 outbreak (9) and the recent detection of a new *Ebolavirus* species, Bombali virus in *M. condylurus* (14) signifying the importance of examining EBOV infection in this species.

Prior studies investigating the susceptibility of bats to EBOV infection have focussed on the use of epithelial or fibroblast cell cultures. These cell types are highly permissive *in vitro*, and *in vitro* susceptibility has not correlated with *in vivo* susceptibility at the level of the organism, which has been shown in the case of *R. aegyptiacus* (35). Rather than epithelial cells and fibroblasts, DCs can be used to address impacts of infection on immune induction such as antigen presentation, T cell activation and stimulation of inflammatory mediators. Investigating DCs may be specifically important for EBOV because poor adaptive immune responses are clearly associated with poor disease outcomes for EBOV patients (4).

In line with previous findings that mouse CD11b^+^ DC (36), monocytes from NHP (37), and cultured DC from human (25) are susceptible to EBOV infection, in our study we observed amplification of viral genome in bat-BMDC cultures. Over the course of infection, genome amplification correlated with increases in expression of two of the six bat host genes tested including CD80, a DC activation marker associated with T-cell priming, and CCL3, a potent chemokine produced by APCs upon stimulation with LPS. Specifically, for EBOV infection CCL3/MIP1α gene expression is associated with EBOV-Makona induced systemic inflammation (37), while CD80 expression is typically inhibited in DC by EBOV infection, culminating in DC impairment (22, 38).

Unlike the archetypal dysregulation observed in human and NHP derived DCs after EBOV infection, our findings suggest bat-BMDC cultures show features consistent with DC activation following EBOV infection. As DC activation is implicated as a key step in immune induction leading to convalescent outcomes for Ebola infected patients, the activation of bat-BMDC could be one immunological factor contributing to the bats inherent ability to develop asymptomatic infections. To further study the significance of these findings more extensive immunological tools and phenotyping panels are required as correlating DC function between species is complex due to the presence of numerous functionally distinct subsets that are often not analogous between different species [reviewed in (39)]. Moreover, the DC surface markers expressed by one species may not be constitutively expressed on the surface of another, for example, CD64 has been used to phenotype human but not mouse macrophages (40). Other markers such as CD11c (41) and XCR1 (42) can also be downregulated over time, in culture or during DC activation.

One limitation of the bat-BMDC culture system described in our study is that the cultures are heterogeneous, likely containing macrophages, which would influence experimental outcomes (32), particularly for the infection studies. The identification of a macrophage-specific marker that is conserved between mammals would be particularly helpful to further delineate the role of macrophages in the infection outcomes we have observed. The absence of species-specific reagents for *M. condylurus* was overcome by use of cross-reactive antibodies and growth factors. Cross-reactive reagents are typically validated via Western blotting, however compatibility with flow cytometry applications was a requirement in this study. Hence, we addressed reagent validity through multiple steps. Firstly, antibodies that bound epitopes with high levels of sequence similarity to *M. condylurus* were selected. These reagents were further tested in binding studies with splenocytes, which confirmed if marker co-expression correlated with established cell phenotypes for other mammals. Furthermore, we tested relevant markers (MHCII) for their ability to be upregulated as a result of agonist stimulation. For markers that had lower levels of sequence homology to *M. condylurus*, where antibody binding was observed, probes were designed to target *M. condylurus* transcripts to confirm correlation of RNA and antibody binding. Of interest, additional markers that we tested on the BMDC cultures did not yield positive cross-reactivity for antibody binding above an isotype control in our study despite considerable levels of amino acid identity to that of a species-relevant amino acid sequence. This was observed for CADM1 (95% identity), CD8α (61.9% identity), XCR1 (74.2% identity), and CD4 (total sequence 55.9% identity, conserved epitope > 95% identity), suggesting the cellular pattern of expression could be atypical for these markers.

Studying the response of DCs from different species to EBOV infection will help pinpoint those likely to act as reservoir hosts based on the capacity of DC to be activated upon infection and induce immunity to facilitate viral clearance. This approach could provide additional information about reservoir host status to the study of EBOV infection in epithelial cells, which are less relevant cell type for determining how the immune system responds to infection. Our findings provide future impetus to further dissect responses of bat-BMDC to EBOV infection by evaluating a broader array of genes with RNAseq or microarrays.

In this study, an *M. condylurus* transcriptome was compiled to develop species-specific immunologic tools and we thereby defined their phylogenetic relationship to other bat species. Microchiropteran genomes available in GenBank include those from species within Miniopteridae, Mormoopidae, Vespertilionidae, Rhinolophidae, and Hipposideridae families (43). Vespertilionidae shares a distant phylogenetic ancestor to Molossidae, the family in which *M. condylurus* is classified, going as far back as the Eocene epoch ~50 million years ago (44). Consistent with this, our blast2go analysis of the transcriptome revealed close evolutionary relationships between *M. condylurus* and *Miniopterus natalensis*, a member of the Vespertilionidae which also inhabits South-East Africa (45). A high number of contigs also aligned to other members of Vespertilionidae, particularly *Myotis brandtii*, which is distributed in Asia and Europe, and fewer contigs to *Myotis davidii* and *Myotis lucifugus*, bats which are found in China and the Americas, respectively. Numerous matches, which did not have a clear phylogenetic basis included those to *Pteropus alecto*, which displayed more sequence matches than other geographically proximal fruit bats, i.e., *Rousettus aegyptiacus*. Outside of the order Chiroptera, top-hit blast matches to human, yak (*Bos mutis*) and porcine (*Sus scrofa*) sequences were identified and are likely due to well-annotated transcriptomes in GenBank for these species. However, this is not the case with the high number of sequence-matches to yak annotations as the number of available annotations for this species is considerably low. These sequences were identified as retrotransposon elements.

One limitation of the transcriptome assembled was the sole use of splenocytes as the source of RNA, which has been shown to create assemblies enriched in metabolic terms akin to kidney and lung profiles (46). Expansion to include other tissues such as lymph node can result in assemblies rich in lymphocyte and leukocyte activation-associated transcripts (47). While the inclusion of other tissues such as lymph node would have been beneficial to the dataset, their collection from healthy microbats is challenging and in order to generate sufficient RNA for sequencing, pooling from several bats would be required.

The transcriptomics data was enlisted to elucidate an appropriate source of growth factors to promote DC differentiation. The use of non-species-matched growth factors is a viable approach that has been harnessed for rabbit (48) and chimpanzee (49) to generate functionally competent DCs that can be matured by TLR ligands, phagocytose FITC dextran and also induce T cell proliferation in mixed leukocyte reaction assays. Our phylogenetic results suggested that bovine or equine growth factors are suitable for *M. condylurus* BMDC propagation. We focussed on equine GM-CSF/IL4, as it has been previously indicated as an ideal supplement to propagate fruit bat BMDCs (50). An advantage of using recombinant supplements rather than a cell-culture derived species-matched method in the form of supernatants (51), is the availability of specific growth factors at well-defined concentrations allowing for accurate dosing as well as dose-optimisation.

The transcriptomics-driven approach we have described here to identify and validate cross-reactive markers and reagents provides an ideal platform that can be utilized to provide further insight into not only how *M. condylurus* but also other bat species can behave as reservoir hosts. Overall, our research highlights that bat-BMDCs are activated as part of the reservoir host response to EBOV infection, which provides evidence and rationale for the development and use of targeted approaches to activate and overcome DC-dysfunction in humans to improve protection against EBOV-disease.

## Methods

### Bat Tissue and Bone Marrow Collection

Animal work and necropsies were performed with the permission of the Laboratoire Central Vétérinair, Laboratoire National d'Appui au Développement Agricole (LANADA), Bingerville, Ivory Coast (No. 05/virology/2016). The animal care and use protocol adhered with the ethics committee of LANADA and National Ethics Committee for Research (CNER). Consent existed to capture the bats from the owners of the residence in Koffikro Village After 2–4 weeks of housing, the bats were anesthetized with Isoflurane (1,214, cp-pharma®) and euthanized by decapitation.

Humeri, femurs and sternum were removed from male bats (weighing between 26 and 35 g each). Muscle and fascia were extirpated with a scalpel and the bones rinsed with 70% ethanol. Bones were transversally sectioned with scissors and the bone marrow was flushed from the bone with Recovery TM Cell Culture Freezing Medium (12648010, Gibco) using a cannula (Braun Sterican® Gr. 18) and collected cells cryopreserved at −80°C. The spleen was removed and single cell suspensions were produced by slicing it into smaller pieces, which were added to cRPMI medium, comprising RPMI-1640 (Sigma, R8758) containing 10% (v/v) FBS, 10 U/ml Penicillin and 10 μg/ml Streptomycin (15140122, ThermoFisher), 40 μM β-Mercaptoethanol (4227.3, Roth), 10 mM HEPES (15630106, ThermoFisher), and 25 U/ml Benzonase (71205-3, Milipore) and gently sieved through a 100 μM strainer (352360, Falcon). Following centrifugation, cells were treated with ACK lysis buffer (A1049201, ThermoFisher) for 6 min at room temperature, washed in cRPMI and resuspended in freezing media, cryopreserved and transported to the RKI in a cryogenic dry shipper.

### Stimulation of Splenocytes

Splenocytes were rested in complete IMDM (cIMDM) (Sigma) containing 10% FBS, 10 U/ml Penicillin and 10 μg/ml Streptomycin, 40 μM β-Mercaptoethanol and 10 mM HEPES. Following centrifugation, splenocytes were re-suspended and 8.5–10 × 10^5^ cells were seeded in 96-well flat-bottom plates. Following overnight culture at 37°C, splenocytes were stimulated with an agonist array; either 10 μg/200 μl lipopolysaccharide (LPS) from Escherichia Coli 011:B4 (tlrl-eblps, Invivogen), 10 μg/200 μl Concanavalin A (ConA) (C0412, Sigma), 1 μg/200 μl Phorbol myristaste acetate (PMA) (tlrl-pma, Invivogen) mixed with 10 μg/200 μl Ionomycin (inh-ion, Invivogen) or transfected with 1 μg/200 μl high molecular weight polyinosinic:polycytidylic acid (poly I:C) (tlrl-pic, Invivogen) using 3 μl Lipofectamine 3000 (L3000001, LifeTechnologies). After 6 h, RNA was extracted with use of TRIzol Reagent (10296028, Invitrogen) according to manufacturer's instructions. The RNA pellet was visualized by the addition of 1 μl of glycogen (10901393001, Roche) and re-suspended in 40 μl RNase free water. RNA yields ranged from 20 to 25 ng/μl as measured with high sensitivity Qubit kits (Q32852, ThermoFisher).

### Rapid Amplification of cDNA Ends (RACE)

5′ and 3′ RACE was performed according to previously published methods with RNA harvested from *M. condylurus* splenocytes (52).

### Transcriptome Sequencing and Analysis

Transcriptomics was performed with splenocytes treated with an agonist array to enrich for transcripts associated with an immune response. To determine the optimal for measuring immune associated transcripts, *M. condylurus* splenocytes were first stimulated with poly IC and IFN-β transcripts detected with endpoint PCR. The mRNA sequence for IFN-β was determined by RACE to design specific primers (Supplementary Figure 1A). We found that relative to a housekeeping gene, cytochrome b, which was detectable at all times examined, IFN-β PCR products were first visualized at 6 h post stimulation (Supplementary Figure 1B).

In a separate experiment splenocytes were treated with the agonist array and RNA for transcriptomic analysis was collected after 6 h. With the aim of generating as many annotations as possible, RNA harvested following all treatments were pooled and analyzed. The transcriptome was sequenced with Illumina HiSeq (Illumina) on rapid run mode as a 250 bp paired end library. One limitation of the data analysis was that the read pairs overlapped in >90% of the sequenced fragments, due to a majority of short DNA fragments in the sequencing library. Hence only the forward reads were used for transcriptome assembly as the reverse reads did not yield additional information.

Quality control and read trimming was performed with QCumber2 (https://gitlab.com/RKIBioinformaticsPipelines/QCumber) filtering out bases with Phred scaled quality of <30 and reads with a minimum sequence length of 50 nt. Trimmed reads are publicly available at NCBI accessible with biosample number SAMN10439459 and project number PRJNA506280. *De novo* assembly quality and annotation were performed of the Oyster River Protocol, which used the Trinity Platform to assemble and annotate transcriptomes from RNAseq data (53). Retained reads from QCumber2 (122,907,600) were subjected to *de-novo* assembly with Trinity (v2.4.0) and Transrate (v1.0.3) was applied to filter out poorly covered contiguous sequences (contigs). Approximately 80,000 contigs were able to be annotated by Uniprot with Trinotate (v3.0.2) and Transdecoder (v4.1.0). Further analysis of trinity annotated contigs was performed with blast2go workbench (54). The amino acid sequences of immune proteins were aligned with use of ClustalW (55) or MUSCLE in Geneious (v10.0.5).

### Bone Marrow DC Cultures

Thawed bone marrow cells (~1 × 10^6^) were cultured in 3 ml cIMDM containing 20 ng/ml equine IL-4 (REIL4I, Fisher Scientific) and GM-CSF (ICT-6379, Biomol) at 37°C and 5% CO_2_ in a 35 mm bacterial petri dish (430588, Corning). On days 2, 4, 7 and 9, cells were fed with fresh medium containing IL-4 and GM-CSF by gently removing 2 ml of supernatant without disturbing the cell monolayer, pelleting the cells at 300 g for 5 min and resuspension of the centrifuged pellet in 2 ml of fresh cytokine supplemented cIMDM. Non-adherent cells from bone marrow cultures were used between day 6 and 10 for phenotypic and functional assays.

### Scanning Electron Microscopy (SEM)

BMDC were flushed from 35 mm petri dishes and allowed to absorb for 3 h on round glass 13 mm coverslips in TC 24-well plates. Coverslips with adherent cells were fixed in buffered (0.05 M HEPES, pH 7.2) 2.5% glutaraldehyde, for 2 h at room temperature and then, gently washed with distilled water prior to post-fixation in 1% OsO_4_ (1 h). After a further wash in distilled water, samples were dehydrated in an ethanol series (30, 50, 70, 90, and 96% for 15 min each and absolute ethanol for 30 min) and critical point dried (CPD 300, Leica, Germany) using carbon dioxide. Finally, the samples were coated with 2 nm gold/palladium using a sputter coater (E5100 Polaron/Quorum Technologies, UK,) and examined in a field emission scanning electron microscope (Leo 1530 Gemini, Carl Zeiss Microscopy, Germany) at 3 kV acceleration voltage and a working distance of 4.2 mm. Signals from an in-lens-SE and an Everhart-Thornley secondary electron detector were mixed (50:50%) for the imaging of all samples.

### Flow Cytometric Staining of Splenocytes and DCs

Splenocytes were thawed and re-suspended in 8 ml of cIMDM prior to staining with antibodies whilst BMDCs were harvested directly from cell cultures. Following one wash in phosphate buffered saline (PBS), cells were stained with BV405 vitality dye (L34964, ThermoFisher) at a 1:100 dilution in PBS for 10 min at room temperature. Following two consecutive washes in PBS containing 10% FBS, cells were resuspended in 0.1 ml 10% normal goat serum. Extracellular staining with the following antibodies in staining buffer (FBØ), which contained 1 mM EDTA, 1% bovine serum albumin (BSA) and 0.1% sodium azide (NaN_3_), was performed prior to fixation; α-bovine-SIRPα (clone DH59B, KingFisher Biotech) diluted 1:50 and detected with a secondary goat α-mouse IgG-PEC7 (clone Poly4053, BioLegend) diluted 1:200, α-mouse-CD11b-PE (clone M1/70, BD Pharmingen) diluted 1:100, α-human-CD44-APC-eFluor®780 (clone IM7, eBioScience) diluted 1:50 and α-human-HLA-DR-BV785 (Clone L243, BioLegend) diluted 1:50. Following fixation with 4% paraformaldehyde for 30 min at 4°C, cells were permeabilised with 0.1% saponin for 20 min at room temperature. Intracellular staining was then performed with the following antibodies; α-mouse-CD3ε (clone 145-2C11, BioLegend) diluted 1:50 and α-mammal-CD79α-APC (clone HM57, Abnova) diluted 1:20. Following 30 min of incubation, cells were washed twice in FBØ and samples were subjected to flow cytometric analysis (CytoFLEX cytometer, Beckman Coulter) and analyzed with CytExpert and Cytobank software.

### Prime-Flow Probes and Staining

Splenocytes 10^6^ per sample were incubated at 37°C for 30 min and then stained with BV405 vitality dye and the surface markers SIRPα and CD11b as per flow cytometry assays. PrimeFlow staining was carried out as per manufacturer instructions (PrimeFlow™ RNA Assay, eBioscience) with the test probes; Type4-RPL13 and Type1-SIRPα. RPL13 was used as a positive control.

### DC Infection and Measurement of Viral RNA Copy Number

BMDCs were utilized for infection studies between days 6 and 10 of culture. Cultures from each bat were infected with EBOV-Makona at a MOI of 3 for 1 h, thereafter BMDCs were washed with fresh media and were seeded into 48-well plates at a concentration of 2.5 × 10^5^ cells/well. Mock uninfected control samples were also set up as a negative control. Cells were harvested at 1, 24, 48, and 72 h post-infection (hpi) and resuspended in RLT Buffer (Qiagen). Supernatants from cultures were also collected into AVL Buffer (Qiagen). RNA was extracted according to the manufacturer's instructions using RNeasy, for cell pellets, and Qiamp Kits, for cell supernatants (Qiagen). EBOV transcripts were quantified using a quantitative PCR (qPCR) assay targeting EBOV-VP30 with use of AgPath-ID One Step RT-PCR Kit (4387391, ThermoFisher). Reactions of 25 μl were formulated by addition of 3 μl of RNA sample to a master mix containing: 400 nm of forward and reverse primer, 200 nm of TaqMan Probe, 1 μl enhancer, 1X buffer, and 1X RT-PCR enzyme mix. The thermal profile included incubation at 15 min 45°C, 10 min 95°C, and 45 cycles of 15 s 95°C and 45 s 60°C. The sample CTs were compared to a standard curve, which was produced using EBOV *in vitro* transcripts of known concentrations that ranged from 10^1^ to 10^7^ copies. This standard curve was used to quantify copy numbers within each sample.

### Development of *M. condylurus*-Specific qPCR Assays

Plasmids encoding *M. condylurus* genes downstream of a T7 promoter were synthesized (Aldevron, USA) for the purposes of *in vitro* RNA synthesis. PCR products were produced by M13 primed standard PCR (PlatinumTaq, ThermoFisher) to yield 50–500 ng/μl of template which was added to HiScribe™ T7 High Yield RNA Synthesis reactions (E2040S, NEB) for transcription carried out overnight at 37°C. Reaction constituents were added according to manufacturer's instructions. *In vitro* transcribed RNA was DNase treated for 30 min (Ambion) and purified with 4M LiCl and two 70% ethanol washes. RNA size was confirmed on a bleach gel containing 0.6% sodium hypochlorite. qPCR primer and probe sequences are listed (Supplementary Table 2) and were designed with Geneious workbench 10.0.5. Primers were further filtered to reduce dimer and heterodimer formation likelihood with use of IDT Oligo Analyzer 3.1. Primer and probe concentrations were optimized for use with Luna Universal Probe qPCR mix (M3004, NEB) to produce an efficiency of >90% within a multiplex configuration. The housekeeping gene RPL13 was included in each multiplex panel as a reference for each assay. Each assay included positive RNA controls to generate a standard curve that was fitted using a non-linear regression analysis (GraphPad Prism, USA). This was then used to determine the slope and y-intercept, which was transformed to calculate copy number/ng in the original RNA sample.

## Data Availability Statement

The datasets generated for this study can be found in the NCBI, biosample SAMN10439459 and bioproject PRJNA506280.

## Ethics Statement

The animal study was reviewed and approved by Ethics committee of Laboratoire National d'Appui au Développement Agricole (LANADA), Bingerville, Cote d'Ivoire.

## Author Contributions

KE, AK, HF, and JP contributed to the conception and the design of the study. MB, AL, EC-H, and AR facilitated data acquisition, provided access to samples, helped carry out experiments, and provided intellectual input into experimental design. JL, OD, and BR assisted with bioinformatics and data analyses. KE and JP wrote the manuscript. All authors contributed to manuscript revision, read and approved the submitted version.

### Conflict of Interest

The authors declare that they have no significant competing financial, professional or personal interests that might have influenced the performance of presentation of the work described in this manuscript.

## Supplementary Material

Supplementary Video 1 Motility of bat-DCs. Bat-BMDC cultures were supplemented with GM-CSF/IL4 and after 10 days the cultures were imaged every 10 s for 20 frames, which were compiled with ImageJ.

## References

[1] Kuhn J Filoviruses: a compendium of 40 years of epidemiological, clinical, and laboratory studies. In: Calisher CH editor. Archives of Virology. Supplementa. Vol. 20. New York, NY: Springer-Verlag Wien (2008). 59–97. 10.1007/978-3-211-69495-4 18637412

[2] Goeijenbier M van Kampen JJ Reusken CB Koopmans MP van Gorp EC. Ebola virus disease: a review on epidemiology, symptoms, treatment and pathogenesis. Neth J Med. (2014) 72:442–8. 25387613

[3] Feldmann H Geisbert TW. Ebola haemorrhagic fever. Lancet. (2011) 377:849–62. 10.1016/S0140-6736(10)60667-8 21084112 PMC3406178

[4] Wauquier N Becquart P Padilla C Baize S Leroy EM. Human fatal zaire ebola virus infection is associated with an aberrant innate immunity and with massive lymphocyte apoptosis. PLoS Negl Trop Dis. (2010) 4:e837. 10.1371/journal.pntd.0000837 20957152 PMC2950153

[5] Younan P Iampietro M Nishida A Ramanathan P Santos RI Dutta M. Ebola virus binding to Tim-1 on T lymphocytes induces a cytokine storm. MBio. (2017) 8:e00845-17. 10.1128/mBio.00845-17 28951472 PMC5615193

[6] Feldmann H Feldmann F Marzi A. Ebola: lessons on vaccine development. Annu Rev Microbiol. (2018) 72:423–46. 10.1146/annurev-micro-090817-062414 30200851 PMC11059209

[7] Jones EM Schuh JA Amman RB Sealy KT Zaki RS Nichol TS. Experimental inoculation of egyptian rousette bats (*Rousettus aegyptiacus*) with viruses of the ebolavirus and marburgvirus genera. Viruses. (2015) 7:3420–3442. 10.3390/v7072779 26120867 PMC4517108

[8] Towner JS Amman BR Sealy TK Carroll SA Comer JA Kemp A. Isolation of genetically diverse Marburg viruses from Egyptian fruit bats. PLoS Pathog. (2009) 5:e1000536. 10.1371/journal.ppat.1000536 19649327 PMC2713404

[9] Mari Saez A Weiss S Nowak K Lapeyre V Zimmermann F Dux A. Investigating the zoonotic origin of the West African Ebola epidemic. EMBO Mol Med. (2015) 7:17–23. 10.15252/emmm.201404792 25550396 PMC4309665

[10] Leroy EM Epelboin A Mondonge V Pourrut X Gonzalez JP Muyembe-Tamfum JJ. Human Ebola outbreak resulting from direct exposure to fruit bats in Luebo, Democratic Republic of Congo, 2007. Vector Borne Zoonotic Dis. (2009) 9:723–8. 10.1089/vbz.2008.0167 19323614

[11] Leroy EM Kumulungui B Pourrut X Rouquet P Hassanin A Yaba P. Fruit bats as reservoirs of Ebola virus. Nature. (2005) 438:575–6. 10.1038/438575a 16319873

[12] Pourrut X Souris M Towner JS Rollin PE Nichol ST Gonzalez JP. Large serological survey showing cocirculation of Ebola and Marburg viruses in Gabonese bat populations, and a high seroprevalence of both viruses in *Rousettus aegyptiacus*. BMC Infect Dis. (2009) 9:159. 10.1186/1471-2334-9-159 19785757 PMC2761397

[13] Swanepoel R Leman PA Burt FJ Zachariades NA Braack LE Ksiazek TG. Experimental inoculation of plants and animals with Ebola virus. Emerg Infect Dis. (1996) 2: 321–5. 10.3201/eid0204.960407 8969248 PMC2639914

[14] Goldstein T Anthony SJ Gbakima A Bird BH Bangura J Tremeau-Bravard A The discovery of Bombali virus adds further support for bats as hosts of ebolaviruses. Nat Microbiol. (2018) 3:1084–9. 10.1038/s41564-018-0227-2 30150734 PMC6557442

[15] Forbes KM Webala PW Jääskeläinen AJ Abdurahman S Ogola J Masika MM. Bombali virus in mops condylurus bat, kenya. Emerg Infect Dis. (2019) 25:955–7. 10.3201/eid2505.181666 31002301 PMC6478230

[16] Ng M Ndungo E Kaczmarek ME Herbert AS Binger T Kuehne AI Filovirus receptor NPC1 contributes to species-specific patterns of ebolavirus susceptibility in bats. Neher RA, editor. Elife. (2015) 4:e11785 10.7554/eLife.11785 26698106 PMC4709267

[17] Speranza E Connor JH. Host transcriptional response to ebola virus infection. Vaccines. (2017) 5:30. 10.3390/vaccines5030030 28930167 PMC5620561

[18] Geisbert TW Hensley LE Larsen T Young HA Reed DS Geisbert JB. Pathogenesis of Ebola hemorrhagic fever in cynomolgus macaques - Evidence that dendritic cells are early and sustained targets of infection. Am J Pathol. (2003) 163:2347–70. 10.1016/S0002-9440(10)63591-2 14633608 PMC1892369

[19] Twenhafel NA Mattix ME Johnson JC Robinson CG Pratt WD Cashman KA. Pathology of experimental aerosol Zaire ebolavirus infection in rhesus macaques. Vet Pathol. (2013) 50: 514–29. 10.1177/0300985812469636 23262834

[20] Stroher U West E Bugany H Klenk HD Schnittler HJ Feldmann H. Infection and activation of monocytes by Marburg and Ebola viruses. J Virol. (2001) 75:11025–33. 10.1128/JVI.75.22.11025-11033.2001 11602743 PMC114683

[21] Wahl-Jensen V Kurz S Feldmann F Buehler LK Kindrachuk J DeFilippis V. Ebola virion attachment and entry into human macrophages profoundly effects early cellular gene expression. PLoS Negl Trop Dis. (2011) 5:e1359. 10.1371/journal.pntd.0001359 22028943 PMC3196478

[22] Gupta M Mahanty S Ahmed R Rollin PE. Monocyte-derived human macrophages and peripheral blood mononuclear cells infected with ebola virus secrete MIP-1alpha and TNF-alpha and inhibit poly-IC-induced IFN-alpha *in vitro*. Virology. (2001) 284:20–5. 10.1006/viro.2001.0836 11352664

[23] Okumura A Pitha PM Yoshimura A Harty RN. Interaction between Ebola Virus Glycoprotein and host toll-like receptor 4 leads to induction of proinflammatory cytokines and SOCS1. J Virol. (2010) 84:27–33. 10.1128/JVI.01462-09 19846529 PMC2798428

[24] Escudero-Perez B Volchkova VA Dolnik O Lawrence P Volchkov VE. Shed GP of Ebola virus triggers immune activation and increased vascular permeability. PLoS Pathog. (2014) 10:e1004509. 10.1371/journal.ppat.1004509 25412102 PMC4239094

[25] Bosio CM Aman MJ Grogan C Hogan R Ruthel G Negley D. Ebola and Marburg viruses replicate in monocyte-derived dendritic cells without inducing the production of cytokines and full maturation. J Infect Dis. (2003) 188:1630–8. 10.1086/379199 14639532

[26] Mohamadzadeh M Chen L Schmaljohn AL. How Ebola and Marburg viruses battle the immune system. Nat Rev Immunol. (2007) 7:556–67. 10.1038/nri2098 17589545

[27] Mahanty S Hutchinson K Agarwal S McRae M Rollin PE Pulendran B. Cutting edge: impairment of dendritic cells and adaptive immunity by Ebola and Lassa viruses. J Immunol. (2003) 170:2797–801. 10.4049/jimmunol.170.6.2797 12626527

[28] Jasper PJ Zhai SK Kalis SL Kingzette M Knight KL. B lymphocyte development in rabbit: progenitor B cells and waning of B lymphopoiesis. J Immunol. (2003) 171:6372–80. 10.4049/jimmunol.171.12.6372 14662835

[29] Schumann J Stanko K Schliesser U Appelt C Sawitzki B Differences in CD44 surface expression levels and function discriminates IL-17 and IFN-γ producing helper T cells. PLoS ONE. (2015) 10:e0132479 10.1371/journal.pone.0132479 26172046 PMC4501817

[30] Holling TM Schooten E van Den Elsen PJ. Function and regulation of MHC class II molecules in T-lymphocytes: of mice and men. Hum Immunol. (2004) 65:282–90. 10.1016/j.humimm.2004.01.005 15120183

[31] Dutertre CA Wang LF Ginhoux F. Aligning bona fide dendritic cell populations across species. Cell Immunol. (2014) 291:3–10. 10.1016/j.cellimm.2014.08.006 25262488

[32] Helft J Böttcher J Chakravarty P Zelenay S Huotari J Schraml BU. GM-CSF mouse bone marrow cultures comprise a heterogeneous population of CD11c^+^MHCII^+^ macrophages and dendritic cells. Immunity. (2015) 42:1197–211. 10.1016/j.immuni.2015.05.018 26084029

[33] Olejnik J Forero A Deflube LR Hume AJ Manhart WA Nishida A. Ebolaviruses associated with differential pathogenicity induce distinct host responses in human macrophages. J Virol. (2017) 91:e00179–17. 10.1128/JVI.00179-17 28331091 PMC5432886

[34] Leendertz SA Gogarten JF Dux A Calvignac-Spencer S Leendertz FH. Assessing the evidence supporting fruit bats as the primary reservoirs for ebola viruses. Ecohealth. (2016) 13:18–25. 10.1007/s10393-015-1053-0 26268210 PMC7088038

[35] Kuzmin IV Schwarz TM Ilinykh PA Jordan I Ksiazek TG Sachidanandam R. Innate immune responses of bat and human cells to filoviruses: commonalities and distinctions. J Virol. (2017) 91:e02471–16. 10.1128/JVI.02471-16 28122983 PMC5375674

[36] Ludtke A Ruibal P Wozniak DM Pallasch E Wurr S Bockholt S. Ebola virus infection kinetics in chimeric mice reveal a key role of T cells as barriers for virus dissemination. Sci Rep. (2017) 7:43776. 10.1038/srep43776 28256637 PMC5335601

[37] Menicucci AR Versteeg K Woolsey C Mire CE Geisbert JB Cross RW. Transcriptome analysis of circulating immune cell subsets highlight the role of monocytes in zaire ebola virus makona pathogenesis. Front Immunol. (2017) 8:1372. 10.3389/fimmu.2017.01372 29123522 PMC5662559

[38] Jin H Yan Z Prabhakar BS Feng Z Ma Y Verpooten D. The VP35 protein of Ebola virus impairs dendritic cell maturation induced by virus and lipopolysaccharide. J Gen Virol. (2010) 91:352–61. 10.1099/vir.0.017343-0 19828757 PMC2831215

[39] Vu Manh TP Bertho N Hosmalin A Schwartz-Cornil I Dalod M. Investigating evolutionary conservation of dendritic cell subset identity and functions. Front Immunol. (2015) 6:260. 10.3389/fimmu.2015.00260 26082777 PMC4451681

[40] Guilliams M Dutertre CA Scott CL McGovern N Sichien D Chakarov S. Unsupervised high-dimensional analysis aligns dendritic cells across tissues and species. Immunity. (2016) 45:669–84. 10.1016/j.immuni.2016.08.015 27637149 PMC5040826

[41] Singh-Jasuja H Thiolat A Ribon M Boissier MC Bessis N Rammensee HG. The mouse dendritic cell marker CD11c is down-regulated upon cell activation through Toll-like receptor triggering. Immunobiology. (2013) 218:28–39. 10.1016/j.imbio.2012.01.021 22445076

[42] Bachem A Hartung E Guttler S Mora A Zhou X Hegemann A. Expression of XCR1 characterizes the Batf3-dependent lineage of dendritic cells capable of antigen cross-presentation. Front Immunol. (2012) 3:214. 10.3389/fimmu.2012.00214 22826713 PMC3399095

[43] Teeling EC Vernes SC Davalos LM Ray DA Gilbert MTP Myers E. Bat biology, genomes, and the Bat1K project: to generate chromosome-level genomes for all living bat species. Annu Rev Anim Biosci. (2018) 6:23–46. 10.1146/annurev-animal-022516-022811 29166127

[44] Peixoto FP Braga PHP Mendes P. A synthesis of ecological and evolutionary determinants of bat diversity across spatial scales. BMC Ecol. (2018) 18:18. 10.1186/s12898-018-0174-z 29890975 PMC5996565

[45] Monadjem A Taylor P Cotterill FPD Schoeman MC Bats of Southern and Central Africa. Johannesburg: Wits University Press (2010).

[46] Shaw TI Srivastava A Chou WC Liu L Hawkinson A Glenn TC. Transcriptome sequencing and annotation for the Jamaican fruit bat (Artibeus jamaicensis). PLoS ONE. (2012) 7:e48472. 10.1371/journal.pone.0048472 23166587 PMC3499531

[47] Lee AK Kulcsar KA Elliott O Khiabanian H Nagle ER Jones ME. De novo transcriptome reconstruction and annotation of the Egyptian rousette bat. BMC Genom. (2015) 16:1033. 10.1186/s12864-015-2124-x 26643810 PMC4672546

[48] Cody V Shen H Shlyankevich M Tigelaar RE Brandsma JL Hanlon DJ. Generation of dendritic cells from rabbit bone marrow mononuclear cell cultures supplemented with hGM-CSF and hIL-4. Vet Immunol Immunopathol. (2005) 103:163–72. 10.1016/j.vetimm.2004.08.022 15621303

[49] Barratt-Boyes SM Henderson RA Finn OJ. Chimpanzee dendritic cells with potent immunostimulatory function can be propagated from peripheral blood. Immunology. (1996) 87:528–34. 10.1046/j.1365-2567.1996.514588.x 8675205 PMC1384129

[50] Schountz T. Immunology of bats and their viruses: challenges and opportunities. Viruses. (2014) 6:4880–901. 10.3390/v6124880 25494448 PMC4276934

[51] Zhou P Chionh YT Irac SE Ahn M Jia Ng JH Fossum E. Unlocking bat immunology: establishment of *Pteropus alecto* bone marrow-derived dendritic cells and macrophages. Sci Rep. (2016) 6:38597. 10.1038/srep38597 27934903 PMC5146944

[52] Chen N Wang WM Wang HL. An efficient full-length cDNA amplification strategy based on bioinformatics technology and multiplexed PCR methods. Sci Rep. (2016) 5:19420. 10.1038/srep19420 26758040 PMC4725349

[53] MacManes MD. The Oyster River Protocol: a multi-assembler and kmer approach for *de novo* transcriptome assembly. PeerJ. (2018) 6:e5428. 10.7717/peerj.5428 30083482 PMC6078068

[54] Gotz S Garcia-Gomez JM Terol J Williams TD Nagaraj SH Nueda MJ. High-throughput functional annotation and data mining with the Blast2GO suite. Nucleic Acids Res. (2008) 36:3420–35. 10.1093/nar/gkn176 18445632 PMC2425479

[55] Thompson JD Gibson TJ Higgins DG. Multiple sequence alignment using ClustalW and ClustalX. Curr Protoc Bioinform. (2003) 2.3.1–22. 10.1002/0471250953.bi0203s00 18792934

