# Supplementary figures and images for: Dendritic Cells Generated From Mops condylurus, a Likely Filovirus Reservoir Host, Are Susceptible to and Activated by Zaire Ebolavirus Infection
Source: Front Immunol. 2019 Oct 11;10:2414. doi: 10.3389/fimmu.2019.02414 (PMC6797855; doi:10.3389/fimmu.2019.02414)

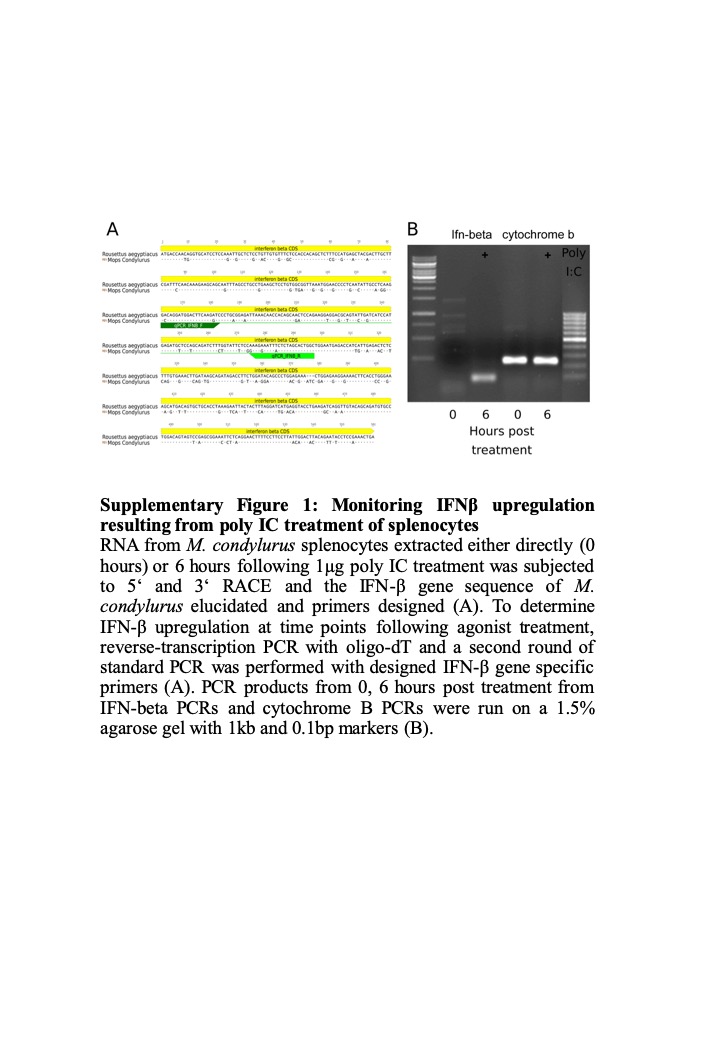

Supplement: Supplementary file 1 [file Image_1.JPEG]

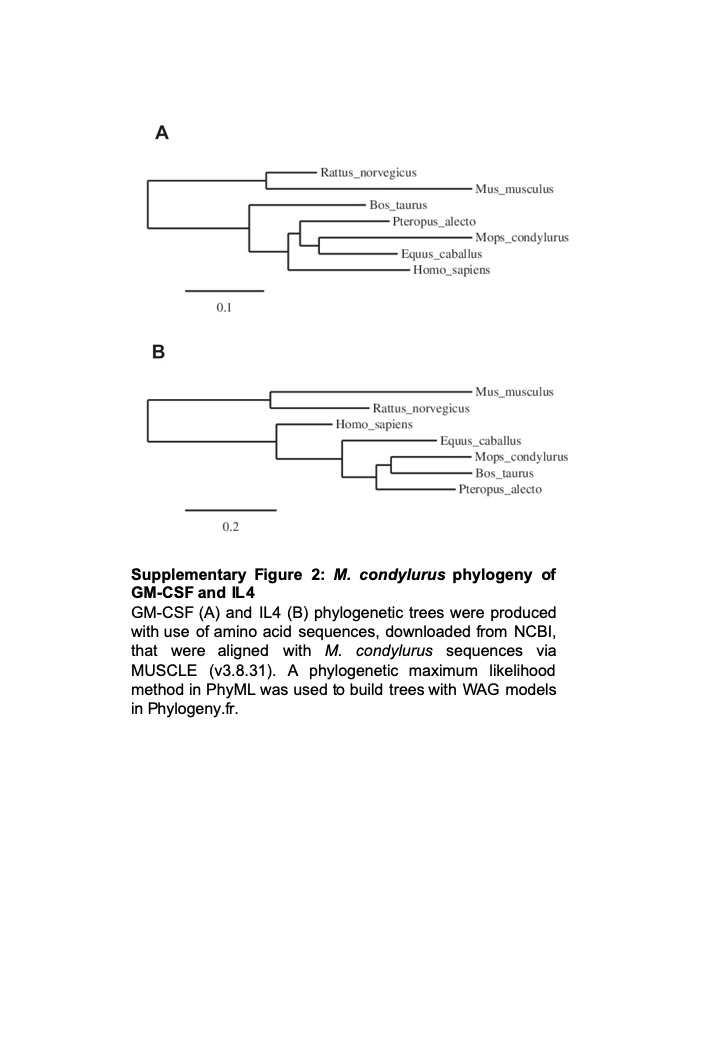

Supplement: Supplementary file 2 [file Image_2.JPEG]
