# Supplementary material for: Dendritic Cells Generated From Mops condylurus, a Likely Filovirus Reservoir Host, Are Susceptible to and Activated by Zaire Ebolavirus Infection
Source: Front Immunol. 2019 Oct 11;10:2414. doi: 10.3389/fimmu.2019.02414 (PMC6797855; doi:10.3389/fimmu.2019.02414)
Supplement: Supplementary file 3 [file Table_1.DOCX]

**Supp. Table 1: *De novo* assembly quality of *M. condylurus***t**ranscriptome**

| **Assembly statistics** |  |
| --- | --- |
| Number of assembled contigs | 547,036 |
| Number of Transrate filtered contigs | 317,247 |
| Number of Uniprot annotated contigs | 80,761 |
| Contig N50 length | 2,345 |
| Median contig length | 888 |
| GC content | 46.0% |
| **BUSCOs detected in eukaryotic lineage** |  |
| Percent complete BUSCOs | 91.1% |
| Percent fragmented BUSCOs | 7.6% |
| Percent missing BUSCOs | 1.3% |
| **NCBI Blast statistics** |  |
| Number of proteins with >80% coverage | 9,316 |
