# Supplementary material for: Dendritic Cells Generated From Mops condylurus, a Likely Filovirus Reservoir Host, Are Susceptible to and Activated by Zaire Ebolavirus Infection
Source: Front Immunol. 2019 Oct 11;10:2414. doi: 10.3389/fimmu.2019.02414 (PMC6797855; doi:10.3389/fimmu.2019.02414)
Supplement: Supplementary file 4 [file Table_2.DOCX]

**Supp. Table 2: qPCR primer, probe and conditions details**

| **Panel** | **Target** | **Primer (5‘-3‘)** | **Probe** | **[Pri/Pro]^a^** | **Eff (%)^b^** |
| --- | --- | --- | --- | --- | --- |
| 1 | MIP1α/CCL3 | **F:**TACCGTCCTTGTCCTCCTCTG  **R:**TTGCCGGTCACAAAATAGTCAAC | TexasRed-TTCGTGTCCCAGCTGATCCCGCGCA-BBQ650 | 0.20/0.15 | 91.50 |
|  | IL4 | **F:**TGAGTGTAGCAGACGTCTTGAC  **R:**TGCTGTAGAGGTTCTTGTCCAG | Cy5-GGCTTGCCCAGAATGCCCCTCCGAA-BHQ3 | 0.20/0.15 | 95.94 |
|  | Eotaxin-1/CCL11 | **F:**AGCTGTGCTCTTCAAGAC **R:**AGGTACTTCATGGCGTCC | FAM-CAGGCCAAGGAGATCTGTGCTGAT-BHQ1 | 0.40/0.30 | 93.17 |
| 2 | CCR7 | **F:**CTTCATCGGCGTCAAGTTTCG **R:**GAGAAGGTGGTAGTGGTCTCG | TexasRed-GAGCAGCTCCGGCAGTGGTCTTCGT-BBQ650 | 0.20/0.15 | 85.70 |
|  | MIP3α/CCL20 | **F:**CAGCTTCGATTGTTGCCTTCG **R:**TTCGGATCTGCACACACAGC | FAM-GCTGTCCAATGAAGCTTGTGACATCGATGC-BHQ1 | 0.20/0.15 | 95.18 |
| 3 | CD184/CXCR4 | **F:**ACAGTCAGAAGCCAAGGAAGC **R:**CATTGGGGTAGAAGCGGTCAC | TexasRed-ACCTGCTCTCCTGTTGGCTATTCCCGA-BBQ-650 | 0.40/0.30 | 91.18 |
|  | CD83 | **F:**GTGAAGGTGGCTTGTTCCCAG **R:**CGGGTGATAGCTGTGCAGATC | Cy5-650GTCCCCTACACCGTCTCCTGGGTCA-BHQ-3 | 0.40/0.15 | 90.94 |
|  | CD80 | **F:**ATTGTGATCCTGGGCCTGC **R:**GCAGACTGATTTCCAAGGTCAC | FAM-CGACGGCGGCAAGTACTCCTGCATCA-BHQ1 | 0.40/0.30 | 95.95 |
| H | RPL13α | **F:**GAAGGCCAAGATCCACTA **R:**GTATGTGCCAATCTTCTTCT | Cy3-AGCTCATGAGGCTACGGAAACAGG-BHQ2 | 0.40/0.15 | 99.87 |

^a^Optimal primer and probe working concentrations (µM) for multiplex assay

^b^Percent efficiency of qPCR across range of 10^2^-10^9^ RNA copies
